# Extending thermotolerance to tomato seedlings by inoculation with SA1 isolate of *Bacillus cereus* and comparison with exogenous humic acid application

**DOI:** 10.1371/journal.pone.0232228

**Published:** 2020-04-30

**Authors:** Muhammad Aaqil Khan, Sajjad Asaf, Abdul Latif Khan, Rahmatullah Jan, Sang-Mo Kang, Kyung-Min Kim, In-Jung Lee

**Affiliations:** 1 School of Applied Biosciences, Kyungpook National University, Daegu, Republic of Korea; 2 Natural and Medical Plants Research Center, University of Nizwa, Nizwa, Oman; Hainan University, CHINA

## Abstract

Heat stress is one of the major abiotic stresses that impair plant growth and crop productivity. Plant growth-promoting endophytic bacteria (PGPEB) and humic acid (HA) are used as bio-stimulants and ecofriendly approaches to improve agriculture crop production and counteract the negative effects of heat stress. Current study aimed to analyze the effect of thermotolerant SA1 an isolate of *Bacillus cereus* and HA on tomato seedlings. The results showed that combine application of SA1+HA significantly improved the biomass and chlorophyll fluorescence of tomato plants under normal and heat stress conditions. Heat stress increased abscisic acid (ABA) and reduced salicylic acid (SA) content; however, combined application of SA1+HA markedly reduced ABA and increased SA. Antioxidant enzymes activities revealed that SA1 and HA treated plants exhibited increased levels of ascorbate peroxidase (APX), superoxide dismutase (SOD), and reduced glutathione (GSH). In addition, heat stress markedly reduced the amino acid contents; however, the amino acids were increased with co-application of SA1+HA. Similarly, inductively-coupled plasma mass-spectrometry results showed that plants treated with SA1+HA exhibited significantly higher iron (Fe^+^), phosphorus (P), and potassium (K^+^) uptake during heat stress. Heat stress increased the relative expression of *SlWRKY33b* and autophagy-related (*SlATG5*) genes, whereas co-application of SA1+HA augmented the heat stress response and reduced *SlWRKY33b* and *SlATG5* expression. The heat stress-responsive transcription factor (*SlHsfA1a*) and high-affinity potassium transporter (*SlHKT1*) were upregulated in SA1+HA-treated plants. In conclusion, current findings suggest that co-application with SA1+HA can be used for the mitigation of heat stress damage in tomato plants and can be commercialized as a biofertilizer.

## 1. Introduction

Temperature plays a vital role in plant growth and development [1]. However, a rise in temperature beyond a threshold level causes irreversible damages to plant growth and development [1,2]. High temperatures affect all developmental stages of plants such as germination, vegetative growth, and gamete, seed and fruit development, resulting in crop yield reduction [3,4]. Lobell et al. [5] reported that high temperatures reduce grain yield per plant by 70% for each 1°C increase in temperature, resulting in 4–14% yield loss in rice. Similarly, You et al. [6] reported a 10% decrease in wheat yield with a 1°C increase in temperature. Furthermore, Intergovernmental Panel on Climate Change (IPCC) reported that India would likely suffer from a 10–40% loss in crop production by 2080–2100 due to heat stress [7].

Heat stress causes various physiological, biochemical, morphological and molecular changes that adversely affect plant growth, biomass, productivity and yield production either individually or in combination with other abiotic stresses [1,2,8]. Leaves and stem scorching, leaf abscission and senescence, root inhibition, shoot development and fruit damage are the physiological injuries observed with an increase in temperature [9,10]. Among the physiological processes, photosynthesis is more sensitive to heat stress and inhabit Photosystem II (PSII), which leads to a decrease in chlorophyll florescence, altering the photosynthetic pigment, foliar expansion and leaf senescence [11–13]. Biochemical changes that can occur in plants include the fluidity of membranes, organization of cellular structure, the structure of amino acids, an increase or decrease in the concentration of metabolites and osmolytes, a decrease in the synthesis of normal proteins, an increase in stress hormones like abscisic acid (ABA), a decrease in defense hormones like salicylic acid (SA), and the production of injurious reactive oxygen species (ROS) and antioxidants. Heat stress often leads to the accumulation of ROS such as superoxide radicals and hydrogen peroxide, which cause oxidative damage and disrupt metabolic homeostasis in plants [14–16]. However, plants activate antioxidant complex systems such as reduced glutathione (GSH), superoxide dismutase (SOD) and other macromolecules (proline, carbohydrates) that protect them from oxidative damages and scavenge excess ROS [14,17]. Molecular changes include the alteration of genes involved in the protection from heat stress. These genes are responsible for the expression of osmoprotectants, detoxifying enzymes and transporters and increases in the regulation of proteins called heat shock proteins (*HSPs*), heat stress transcription factors (*HSFs*), stress-induced proteins or stress proteins that are expressed and play key roles in conferring stress tolerance when plants are exposed to any stressors [18–20].

Heat stress tolerance is also achieved through genetic engineering, breeding programs, tissue culture, maturation and chemical fertilizer application, which are time consuming, costly and have adverse effect on the environment [20–23]. One alternative and ecofriendly approach for the improvement of agricultural crop production to ameliorate the negative effects of high temperature is the use of plant growth-promoting bacteria and chemicals such as humic substances. This biological technique is extremely popular and is broadly accepted all over the world [24–29]. Humic acid (HA) is a heterogeneous mixture of many compounds that enhance plant growth under normal and abiotic stress conditions [28–31]. The use of plant growth promoting bacteria is another approach for ameliorating the negative effects of abiotic stress (21–24). In the last couple of decades, a number of different researchers reported the use of plant growth-promoting bacteria (PGPB) for enhancing tolerance to heat stress in plants such as sorghum [26], chick pea [32], wheat [27,33], tomato [34], and potato [35]. Furthermore, PGPBs have the ability to produce phytohormones that help make plants tolerant against heat stresses by enhancing biofilm formation as well as reducing ABA level and HSP accumulation [24,26,27,32,33,36]. Previous reports indicated that significant changes in the root architecture in non-leguminous plants induced by humic substances may favor the fitness of bacteria-plant interactions, thus resulting in a significant increase in bacteria attachment and survival on plant surfaces as well as endophytic colonization [37–39]. Regarding the mutual interactions between plant roots and microorganisms, previous studies [39–41] reported that heat stress is a key factor in determining soil fertility as a candidate vehicle for PGPB and co-inoculation could be an excellent approach. Other studies [40,42,43] indicated a further benefit of the interaction between microorganisms and organic matter through biological substrate enrichment.

Tomato is one of the most popular and widely consumed vegetables grown worldwide. It is the second most popular vegetable after potato and is considered a good source of dietary minerals, vitamins, lycopenes and other essential nutrients [44,45]. Although tomato has the potential to be cultivated in every location throughout the world, high temperatures above its optimum temperature decrease growth, biomass and yield. According to Abdellatif et al. [46], high temperature affects all stages of tomato plants from the germination to reproductive phases and affects several physiological and biochemical processes dealing with final yield reduction. Canellas and Olivares [47] reported the combined application of PGPB and HA on several crops. Similarly, Busato et al. [48] applied a microbial suspension and a humic substance to plant substrates to promote seedling adaptation to stressful environments. Pishchik et al. [49] reported the impact of combined application of microbes and HA on tomato plants. However, least information are available on the combined effects of PGPB specifically endophytes and HA on tomato seedlings under heat stress conditions. Hence, in the present study, it was aimed to understand the combined effects of thermotolerant SA1 an isolate of *Bacillus cereus* and HA on tomato seedling’s growth and physiological changes at the biochemical, and molecular levels under heat stress.

## 2. Materials and methods

### 2.1. Isolation and screening of bacterial strains

Four plant samples (*Artemisia princeps* Pamp, *Chenopodium ficifolium* Smith, *Oenothera biennis* L. and *Echinochloa crus-galli*) were collected for the isolation of endophytic bacteria from the sand dunes at Pohang beach (latitude 36°7′56.2′′N, longitude 129° 23′55.1′′E) in the Republic of Korea. The method described by khan et al [50,51] was used for the isolation and screening of bacterial endophytes from the roots of the above mentioned plants.

Prior to bioassay assessment and molecular identification, pure cultures of the selected isolates were screened for the production of indole-3-acetic acid (IAA), siderophores and phosphate solubilization potential. Salkowski reagents were used for the initial confirmation of IAA production according to the protocol developed by Patten and Glick [52]. The detailed method described by Katznelson and Bose [53] and Khan et al [51] was assessed for phosphate solubilization potential. For siderophore production, the commonly used method introduced by Schwyn and Neilands [54] was followed. For bioassay assessment, bacterial isolates that revealed prominent plant growth-promoting traits in the initial screening were also screened on *Waito-C* (gibberellin [GA]-deficient) rice seedlings.

### 2.2. Molecular identification and screening for thermotolerant bacteria

Two different methods were used for the identification of thermotolerant bacteria. In the first method, multiple plant growth-promoting traits-producing bacteria were streaked on Luria-Bertani (LB) agar plates and kept at 30, 35, and 40°C for 6 days. The growth of the selected isolates was checked on a daily basis. In the second method, isolates were grown in LB broth media for 6 days consecutively. The culture was grown on a rotatory shaker at 35, 40, and 45°C. The growth of all the isolates was recorded using a spectrophotometer at 600 nm for 6 days. For molecular identification, rRNA was isolated using the detailed method presented by Sambrook and Russell [55]. Specific primers for 16S rDNA, 1492 Reverse (5′-CGG (T/C) TA CCT TGT TAC GAC TT-3′) and 27 Forward (5′-AGA GTT TGA TC (C/A) TGG CTC AG-3′) were used and amplified according to the protocol described by Khan et al. [50]. For the determination of nucleotide sequence homology for the selected isolates, the BLAST NCBI tool was used, while MEGA 6.1 was used for phylogenetic analysis as suggested by Tamura et al. [56].

### 2.3. *In-vitro* IAA, GA and organic acid quantification of isolate SA1

For the quantification of IAA, GA and organic acids, isolate SA1 was grown on LB media for 3 days and centrifuged at 5000_X_g for 15 min. Following the method developed by Khan et al. [57], the culture broths of selected isolates were analyzed for IAA production. Similarly, for the extraction and quantification of the GA content, the method described by Khan et al [58] was followed and the data was calculated in nano-grams per milliliter. Organic acids were determined using Kang et al.’s method [59]. In brief, the culture broth of the bacterial isolate was centrifuged and the supernatant was filtered through 0.22-μm SmartPor Syringe Filter (P/N SPU0213-1). A total of 10 μL of the filtrate sample was injected into a high-performance liquid chromatography system (HPLC; Waters 600, Milford, MA, USA). For determination of the organic acids, the retention time and peak areas were compared with standards from Sigma-Aldrich, USA. All of the samples were analyzed in triplicate.

### 2.4. Plant-microbe growth and heat stress conditions

Vir Yegwang tomato seeds were purchased from Danong Co and surface sterilize with 70% EtoH followed by 2.5% NaOH and rinsed with deionized distilled water [60]. After surface sterilization, the seeds were germinated in an incubator (28°C). After 20 days of germination in the trays, uniform seedlings were selected for further processing. Plastic pots (440×270×195mm) were filled with three-time autoclaved field soil (M2 greenhouse at Kyungpook National University) and used for the growth of tomato. After 1 week of transplantation, 1L of freshly diluted bacterial culture(10^9^ CFU/mL) was inoculated to each pot; this was repeated further two time after 5 days, similarly 500mg/L of humic acid was used [61,62], while autoclaved double distilled water were used for control tomato plants. At V3 stage plants were exposed to heat stress and plants sample were collected at 15 days. The experimental design was as follows: (a) Control (normal tomato), (b) Tomato with Isolate SA1, (c) Tomato with HA, (d) Tomato with SA1+HA, (e) heat stress (37°C), (f) heat stress with SA1, (g) heat stress with HA, (h) heat stress with SA1+HA in a growth chamber. Normal tomato plants were subjected to a 24-hour cycle at 28°C for 14 hours and 25°C for 10 hours with a relative humidity of 60 to 70% while the heat stress group was subjected to a 24-hour cycle at 37°C for 14 hours and 30°C for 10 hours with a relative humidity of 60 to 70%. Upon stress completion, growth attributes such as root/shoot length, biomass (fresh and dry weight) were recorded. The plants (shoot) were immediately harvested in liquid nitrogen and stored at -80 °C until further biochemical analyses. For chlorophyll estimation, a chlorophyll fluorometer (FIM 1500, ADC Bioscientific Ltd, UK) was used to measure chlorophyll fluorescence. The collected data were used for photosystem II (Fv/Fm) calculation as reported by Genty et al. [63].

### 2.5. Plant endogenous phytohormone quantification

The plant samples were subjected to endogenous phytohormone analysis and quantification under a controlled environment. Endogenous ABA was quantified according to the detailed method described by Asaf et al. [64] and Khan et al [65]. The pulverized shoot plant samples were treated with 30 mL of extraction solution containing 95% isopropanol, 5% glacial acetic acid, and 20 ng of [(±)– 3,5,5,7,7,7–d^6^]–ABA. The extracts were dried and methylated by adding diazomethane for GC-MS SIM (6890 N network GC system, and 5973 network mass-selective detector; Agilent Technologies, Palo Alto, CA, USA) analysis. On the other hand, SA was quantified following the method of Jan et al. [66]. Freeze-dried aerial parts were quantified using a HPLC system equipped with a fluorescence detector (excitation and emission at 3005 and 365 nm, respectively; Shimadzu RF-10AXL; Shimadzu, Kyoto, Japan) and fitted with a C18 reverse phase HPLC column (particle size 5 m, pore size 120 Å; HP Hypersil ODS; Waters Co., Milford, MA, USA) at a defined flow rate (1.0 ml/min).

### 2.6. Antioxidant analysis

LPO was analyzed according to the method of Bilal et al. [67]. Plant shoots were ground with liquid nitrogen, and 10 mM phosphate buffer (pH 7) was added. The reaction mixture was prepared by adding 0.2 ml of 8.1% sodium dodecyl sulfate, 1.5 ml of 20% acetic acid (pH 3.5), and 1.5 ml of 0.81% thiobarbituric acid to the supernatant, heating in boiling water for 60 min, and immediately cooling on ice to room temperature. Then, 5 ml of butanol:pyridine solution (15:1 v/v) was added. The upper layer was removed, and the resulting pink color was measured at 532 nm using a spectrophotometer. APX activity was measured following the method of Kim et al [68]. The plant sample was added to the reaction mixture (50 mM potassium phosphate pH 7, 0.5 mM ascorbate, 0.1 mM hydrogen peroxide, and 0.1 mM EDTA), and the decrease in absorbance was measured from 10 to 30 s at 290 nm using a spectrophotometer. The method of Khan et al. [69] was adapted for SOD activity assay. In brief, leaf samples (100 mg) were homogenized with 0.01 M phosphate buffer at pH 7.0 and centrifuged (17,000 × *g* for 15 min at 4°C). The supernatant was used as a crude enzyme extract and passed through a reaction mixture containing Tris-HCl buffer (2 ml) pH 8.2, double-distilled water (2 ml), and 2 mM pyrogallol (0.5 ml). The absorption of the assay mixture and blank (lacking pyrogallol or tissue homogenate) was measured at 470 nm using a spectrophotometer (Shimadzu, Kyoto, Japan) at 180 s intervals. The data are expressed as units/mg of protein. To determine the reduction in GSH concentration, each sample (500 mg) was treated with 2 ml of 10% trichloroacetic acid and centrifuged at 10,000 rpm for 15 min at 4°C. The resulting supernatant (1 ml) was combined with 0.5 ml of Ellman’s reagent and 3 ml of 15 mM sodium phosphate buffer (pH 7.4) and incubated for 5 min at 30°C. The absorbance was measured at 412 nm using a spectrophotometer [70,71]. All experiments were performed three times.

### 2.7. RNA extraction, cDNA synthesis, and qRT-PCR analysis

The protocol developed by Chan et al. [72] was adopted with some modifications. The total RNA was extracted from the crushed leaves using TRIzol™ reagent. The quality of RNA was examined by nanodrop. cDNA was synthesized using qPCRBIO cDNA Synthesis Kit from PCRBIOSYSTEMS. Quantitative real-time RT-PCR (qRT-PCR) was performed using qPCRBIO SYBR Green Kit from PCRBIOSYSTEM, using synthesized (1μl) cDNAs as templates and the gene-specific primers [73]. To normalize the level of relative expression of each gene, actin was used for each reaction and the expression level was calculated in control plants relatively with other treated plants. The reaction was performed in a 20μl volume containing 7μl ddH2O, 1μl primer, 10μl SYBR green and 1μl cDNA and the reaction was repeated trice (S1 Table). A total sample volume of 50 μl was subjected to the following conditions: initial denaturation at 94°C for 5 min, 40 cycles of denaturation at 94°C for 30 s, annealing at 58°C for 30 s, extension at 72°C for 1 min, and final extension at 72°C for 5 min.

### 2.8. Amino acid quantification

For amino acid analysis, the detailed method developed by Kang et al. [60] was followed. Freeze-dried plant samples (100 mg) were hydrolyzed under vacuum in 6 N HCl in an ampulla tube for 24 h at 110°C followed by 80°C for 24 h. The solid residue was homogenized in 0.02 N HCl and filtered with a 0.45-m filter membrane. The amino acids were analyzed using an atomic amino acid analyzer (L-8900, Hitachi, Japan). The concentrations were measured by comparing with specific standards.

### 2.9. Statistical analysis

The experiments were performed in triplicate and the resulting products were used for further analysis. The differences among the mean values were compared with Duncan Multiple Range Test (DMRT) using the SAS (9.2, Cary, NC, USA) statistical software program. Graphical presentation was performed in Graph Pad Prism.

## 3. Results

### 3.1. Isolation, screening for indole-3-acetic acid, phosphate solubilization, siderophore production and bioassay assessment

From the roots of *O*. *biennis*, *C*. *ficifolium*, *A*. *princeps* and *E*. *crus-galli*, a total of 59 endophytic strains were isolated (S2 Table). These isolates were screened for different plant growth-promoting traits (PGP); i.e. IAA, GA, phosphate solubilization and siderophore production. Only 13 isolates showed multiple plant growth promoting (PGP) traits (S2 Table). These isolates were subsequently applied to *Wito-C* (gibberellin [GA]-deficient) rice and only eight isolates induced a significant increase in the growth attributes (root and shoot) compared to the other isolates and control plants.

### 3.2. Identification of thermotolerant bacteria

All the selected isolates were examined for their ability to grow at 25°C, 30°C, 35°C, 40°C and 45°C on both solid and liquid broth media. The results showed that the isolates grew at 40°C on solid as well as on broth medium. However, increasing the temperature to 45°C inhibited the growth of all isolates, and only SA1 showed tolerance to heat stress (S1 and S2 Figs). Therefore, isolate SA1 was selected for further investigation. For molecular identification and phylogenetic analysis of isolate SA1, 16S rRNA was amplified, sequenced and compared to the database of known 16S rRNA genes. Our results revealed that SA1 exhibited a high level of 16S sequence identity (99%) with *B*. *cereus*. The SA1 16S rRNA sequence was submitted to NCBI with GenBank accession no MH032605 (S3 Fig).

### 3.3. *In-vitro* IAA, GA and organic acid production of the bacterial isolates

**Fig 1:**
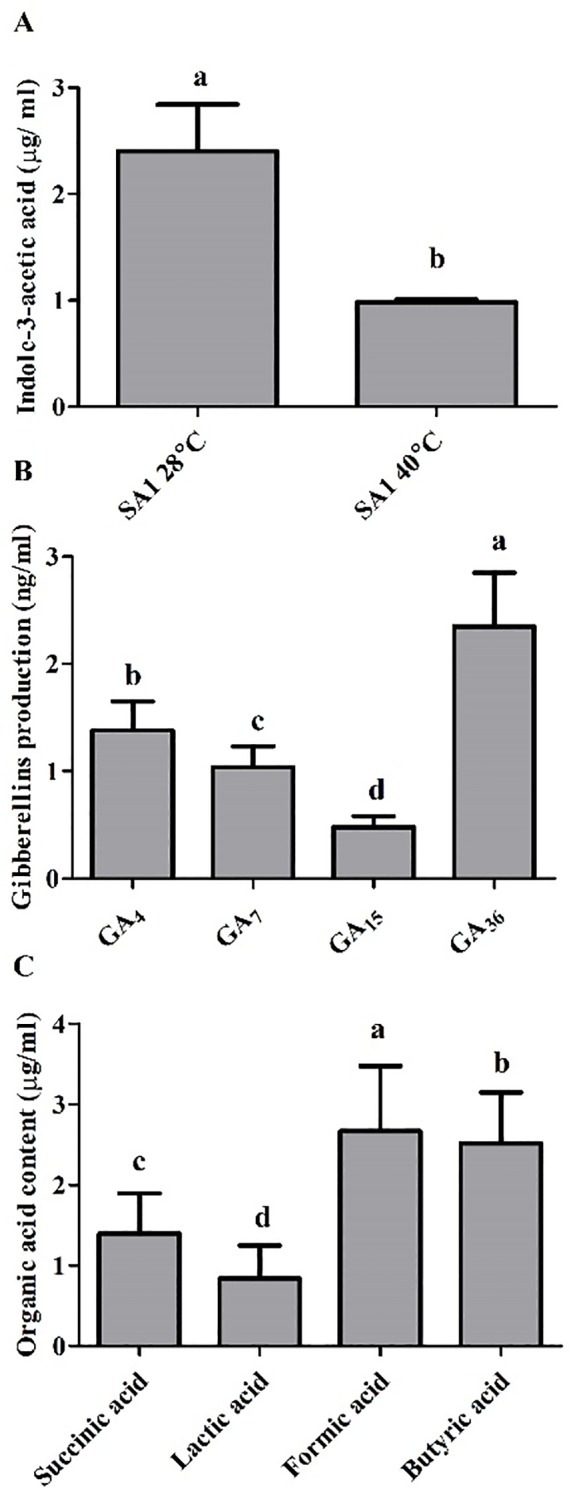
Quantification of indole-3-acetic acid (IAA), gibberellins (GAs) and organic acids produced by SA1 an isolate of *Bacillus cereus*. (A) IAA content was detected in culture broth (CB) of SA1, grown at 28°C and 40°C, through Gas chromatography–mass spectrometry (GC/MS-SIM) (B) GC/MS-SIM analysis and quantification of different GAs in CB of SA1 and its comparison with the internal standard. (C) Organic acid content analysis, detection and quantification through high-performance liquid chromatography system (HPLC) in relation to their respective standards. Each data is the mean of three replicates. Error bars represent standard errors. The bars presented with different letters are significantly different from each other as evaluated by Duncan’s Multiple Range Test (DMRT) analysis.

The culture filtrate (CF) of isolate SA1 was quantified for phytohormones (IAA and GAs) using GC/MS and for organic acids using HPLC. The selected isolate SA1 produced significant amounts of IAA and GA (bioactive and non-bioactive) (Fig 1A and 1B). Organic acid analysis revealed that the CF of SA1 contained lactic acid, butyric acid, formic acid and succinic acid (Fig 1C).

### 3.4. Ameliorative effects of isolate SA1 and HA against heat stress

**Fig 2:**
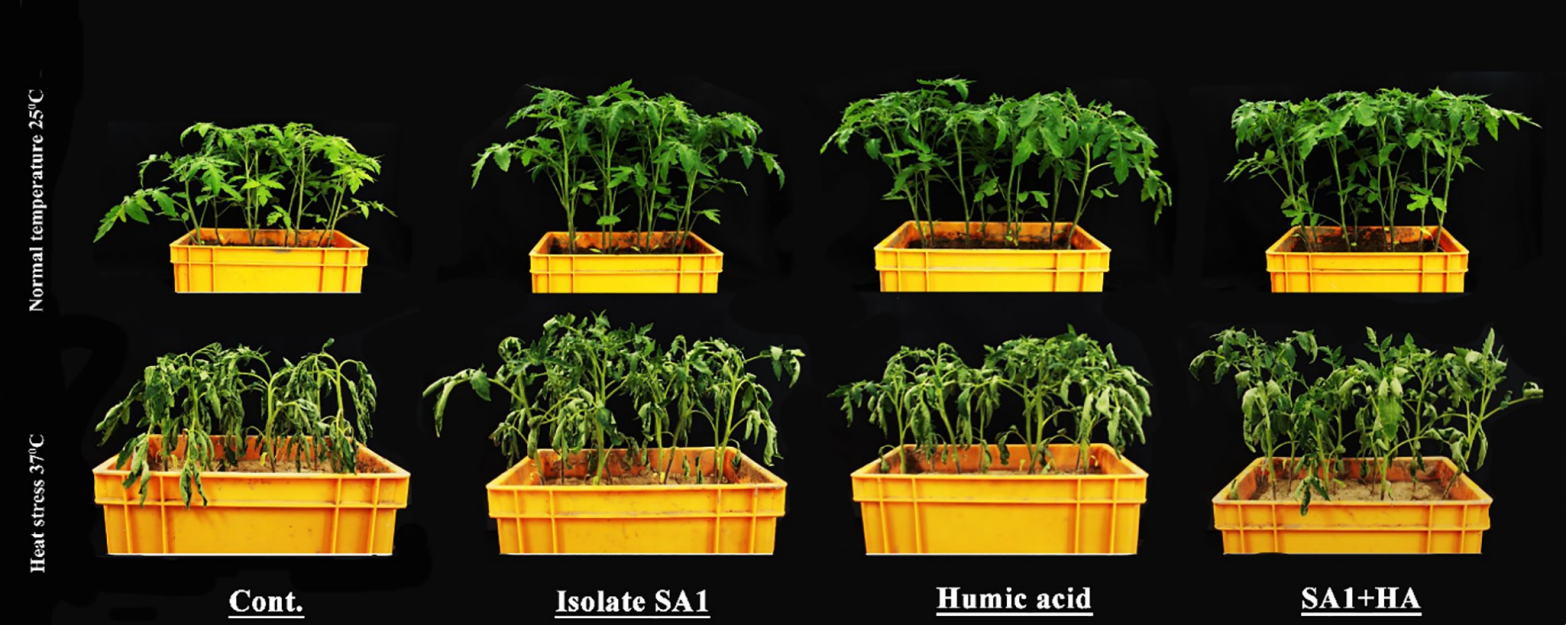
Effects of selected SA1 an isolate of *Bacillus cereus* (SA1), humic acid (HA) and combined SA1+HA application on the growth of tomato plants under normal and heat stress.

Heat stress adversely affected the growth attributes of tomato; however, application of isolate SA1 and HA promoted the growth and biochemical parameters of tomato plants and significantly enhanced their thermotolerance. Under normal growth conditions, the application of HA, isolate SA1 and combined SA1+HA application significantly enhanced the shoot length by 22, 27 and 41% and the root length by 9, 6 and 19%, as well as the fresh weight (80–93%) and dry weight (61%-67%) when compared with normal control tomato plants (Table 1; Fig 2). Similar significant increases were observed in the root fresh and dry weight compared to the control plants (Table 1). However, when tomato plants were subjected to heat stress, combined application of SA1+HA significantly mitigated the adverse effects, with significantly higher growth attributes compared with sole SA and sole HA-inoculated plants and the control (stressed) plants (Fig 2). Heat stress significantly reduced the root and shoot lengths by 36 and 35% in control (heat stressed) plants, whereas combined application of HA+SA1 significantly enhanced the lengths up to 34 and 25% compared with sole SA1 and HA-treated plants. On exposure to heat stress, combined SA1+HA-treated plants displayed a significant restoration of fresh weight (1.5 fold) and dry weight (single fold) (Table 1). Similarly, chlorophyll fluorescence results showed a significant increase in SA1+HA-treated plants compared with control (stressed) plants (Table 1).

**Table 1:** Effect of SA1 an isolate of *B*. *cereus*, humic acid (HA) and SA1+HA on tomato plants under normal and heat stress.

	Control	SA1	HA	HA+SA1
**Normal Temperature**				
**SL (cm)**	32.66±1.7^c^	40.33±1.3^b^	41.83±3.7^b^	46.5±5.3^a^
**RL (cm)**	14.4.±1.5^c^	15.7±1.5^b^	15.3±1.6^bc^	17.3±1.7^a^
**SFW (g)**	23.96±1.7^b^	43.26±2.3^a^	44.66±3.3^a^	46.33±2.3^a^
**SDW (g)**	2.7±0.9^b^	4.36±0.9^a^	4.41±0.7^a^	4.53±0.6^a^
**RFW(g)**	16.80±1.1^c^	21.19±1.9^b^	22.26±2.1^b^	30.08±1.6^a^
**RDW(g)**	2.88±0.3^c^	3.03±0.1^bc^	3.54±0.4^b^	4.23±0.3^a^
**CF (Fv/Fm)**	0.81±0.02^a^	0.81±0.03^a^	0.82±0.04^a^	0.84±0.04^a^
**Heat stress**				
**SL (cm)**	21.16±1.7^b^	26.66±2.5^a^	26.33±1.7^a^	28.5±3^a^
**RL (cm)**	9.2±1.4^c^	10.3±2.1^b^	10.8±1.4^b^	11.5±2.1^a^
**SFW (g)**	12.3±1.1^c^	22.33±1.8^b^	21.13±1.6^b^	29.06±1.7^a^
**SDW (g)**	1.54±0.2^c^	2.61±0.3^b^	2.32±0.4^ab^	2.96±0.2^a^
**RFW(g)**	11.83±1.6^b^	14.39±0.8^ab^	12.47±1.3^ab^	15.0±1.6^a^
**RDW(g)**	1.1±0.2^c^	1.5±0.2^b^	1.7±0.1^ab^	1.9±0.1^a^
**CF (Fv/Fm)**	0.6±0.02^c^	0.68±0.104^b^	0.73±0.05^b^	0.77±0.08^a^

SL = Shoot length, SFW = shoot fresh weight, SDW = shoot dry weight, RL = root length, RFW = root fresh weight, RDW = root dry weight and CF = Chlorophyll fluorescence. Each data point is the mean of three replicates. Error bars represent standard errors. The bars presented with different letters are significantly different from each other as evaluated by Duncan’s Multiple Range Test (DMRT) analysis

### 3.5. Quantification of plant endogenous phytohormones

**Fig 3:**
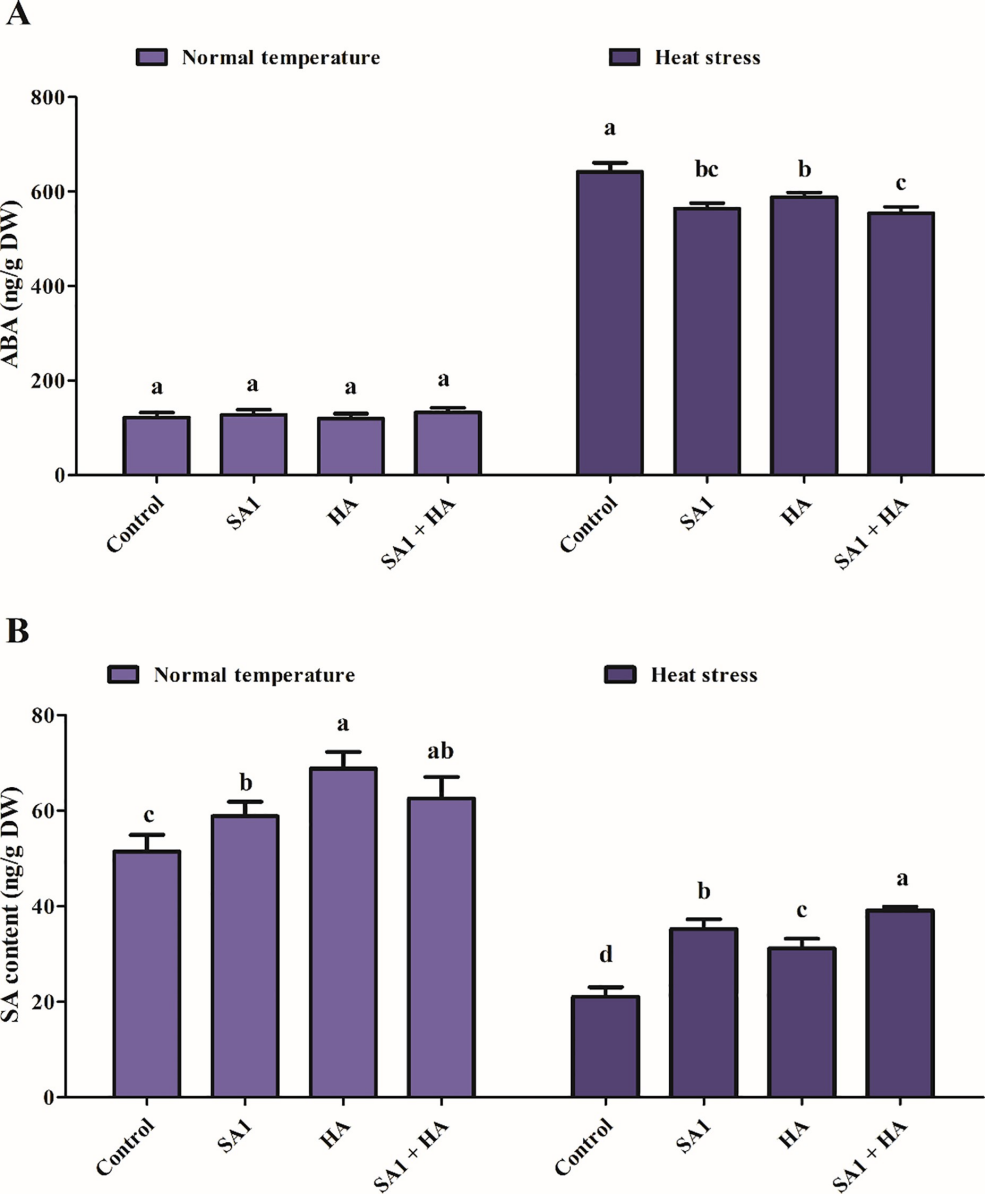
(A) Endogenous abscisic acid (ABA) and (B) salicylic acid (SA) quantification in tomato plants inoculated with SA1 an isolate of *Bacillus cereus* (SA1), humic acid (HA) and combined SA1+HA application. Each data point is the mean of at least three replicates. Error bars represent standard errors. The bars presented with different letters are significantly different from each other as evaluated by Duncan’s Multiple Range Test (DMRT).

A significant increase in ABA content and a decrease in SA content were observed after exposure to heat stress. The endogenous ABA content showed a significant increase up to three-fold compared to control tomato plants (Fig 3A). In contrast, application with thermotolerant isolate SA1, HA and combined SA1+HA remarkably reduced the ABA content (1.5–2 fold). Similarly, both unstressed (normal temperature) and stressed (heat stressed) tomato plants were subjected to SA analysis. The results showed that in contrast to the endogenous ABA level, a decrease in SA content was observed in tomato plants exposed to heat stress. The endogenous SA content was significantly reduced from 48%; however, inoculation with SA1, HA and combined SA1+HA mitigated the heat stress and increased the SA content from 26.1 to 58.2% (Fig 3B).

### 3.6. Modulation of tomato antioxidant system under heat stress

**Fig 4:**
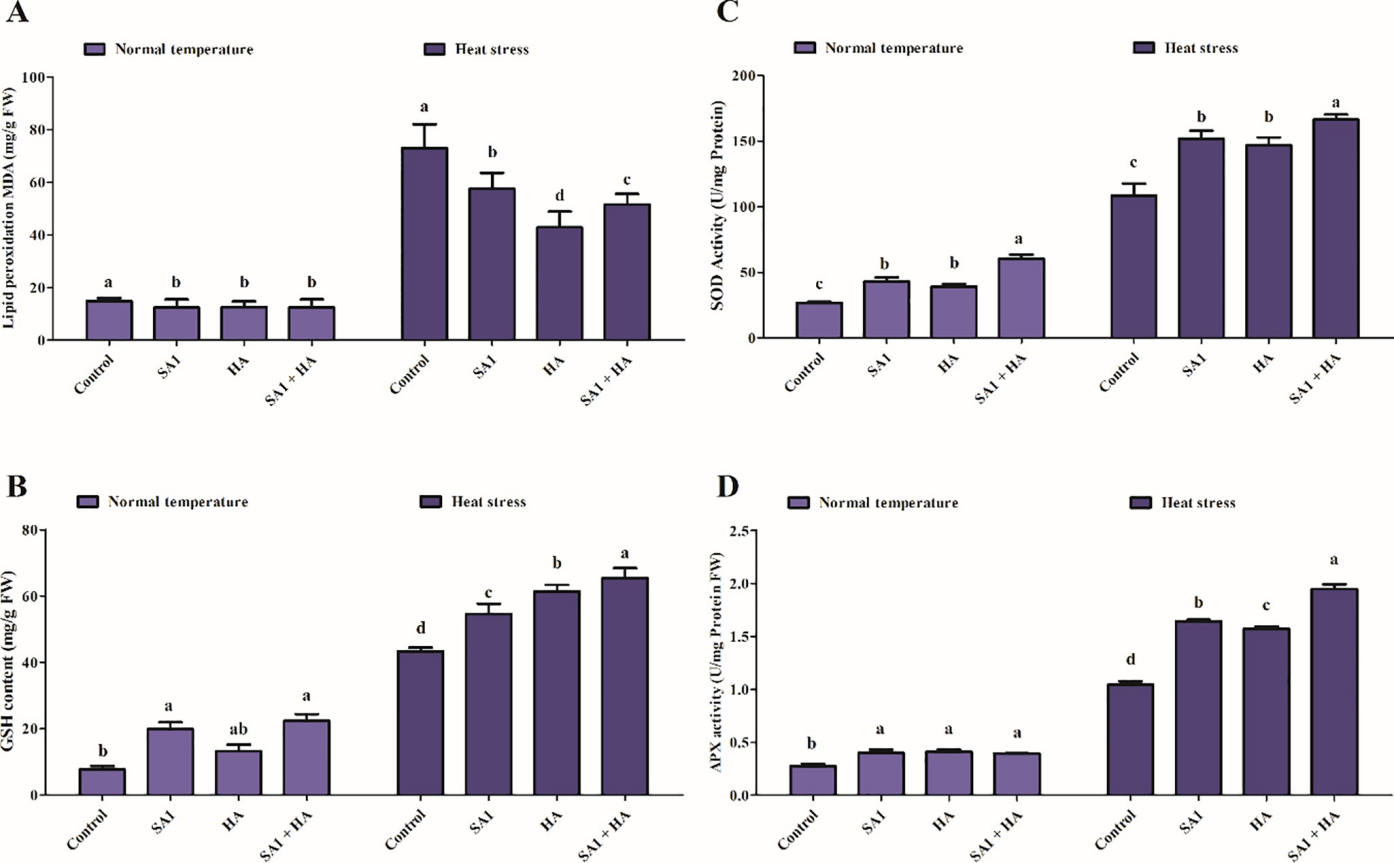
Effect of SA1 an isolate of *Bacillus cereus* (SA1), humic acid (HA) and combined SA1+HA application on different antioxidants; (A) Lipid peroxidation (MDA), (B) Reduced glutathione (GSH), (C) Superoxide dismutase (SOD) and (D) Ascorbic peroxidase (APX) contents in tomato plants under normal and heat stress. Each data point is the mean of three replicates. Error bars represent standard errors. The bars presented with different letters are significantly different from each other as evaluated by Duncan’s Multiple Range Test (DMRT) analysis.

To assess the extent of lipid peroxidation in tomato due to heat stress-induced oxidative stress, the malondialdehyde (MDA) content was investigated (Fig 4). Heat stress activates MDA production, which in turn leads to the induction of lipid peroxidation. In the current study, not significant difference was observed in MDA content in the sole SA1, HA and SA1+HA treated tomato leaf tissues under normal temperatures (Fig 4A). However, when the tomato plants were exposed to heat stress, an increase in the MDA levels (385%) was observed compared to the combined application of SA1+HA (245%) and sole application of SA1 (285%) and HA (187%). The MDA content in HA-treated plants was significantly reduced under heat stress conditions relative to the MDA contents of the sole SA1-inoculated plants and combined SA1+HA-treated tomato plants. To further elucidate the oxidative stress mitigation, the GSH content in plants was examined. Under normal temperatures, SA1, HA and combined SA1+HA-treated plants showed significantly higher GSH content (90%-187%) compared with the control tomato plants (Fig 4B). Heat stress induced an increase in GSH content; however, a significantly higher amount of reduced GSH was generated in SA1 (607%), HA (695%) and combined SA1+HA-treated (740%) plants compared to the heat stressed control tomato plants (461%). In terms of SOD activity, the results indicated that sole application of SA1, HA and combined SA1+HA differentially regulated the SOD activities under normal and heat stress conditions. Under the normal temperature, no significant difference was observed in sole SA1 (469%), HA (450%) and combined SA1+HA-treated (523%) tomato plants. However, under heat stress, significant enhancement in the SOD activity of the combined SA1+HA-treated tomato plants was observed compared with the sole SA1 and HA-treated tomato plants and heat stress control (307%) plants (Fig 4C). Ascorbate peroxidase results showed a significant difference in tomato plants treated with sole or combined SA1+HA (41–49%) compared with control plants. However, under heat stress, significant enhancement in APX was observed in the combined SA1+HA-treated (608%) tomato plants compared with the sole SA1 (492%) and HA (470%) treated tomato plants and heat stressed control plants (283%) (Fig 4D).

### 3.7. Role of SA1 and HA in ion uptake during heat stress

**Fig 5:**
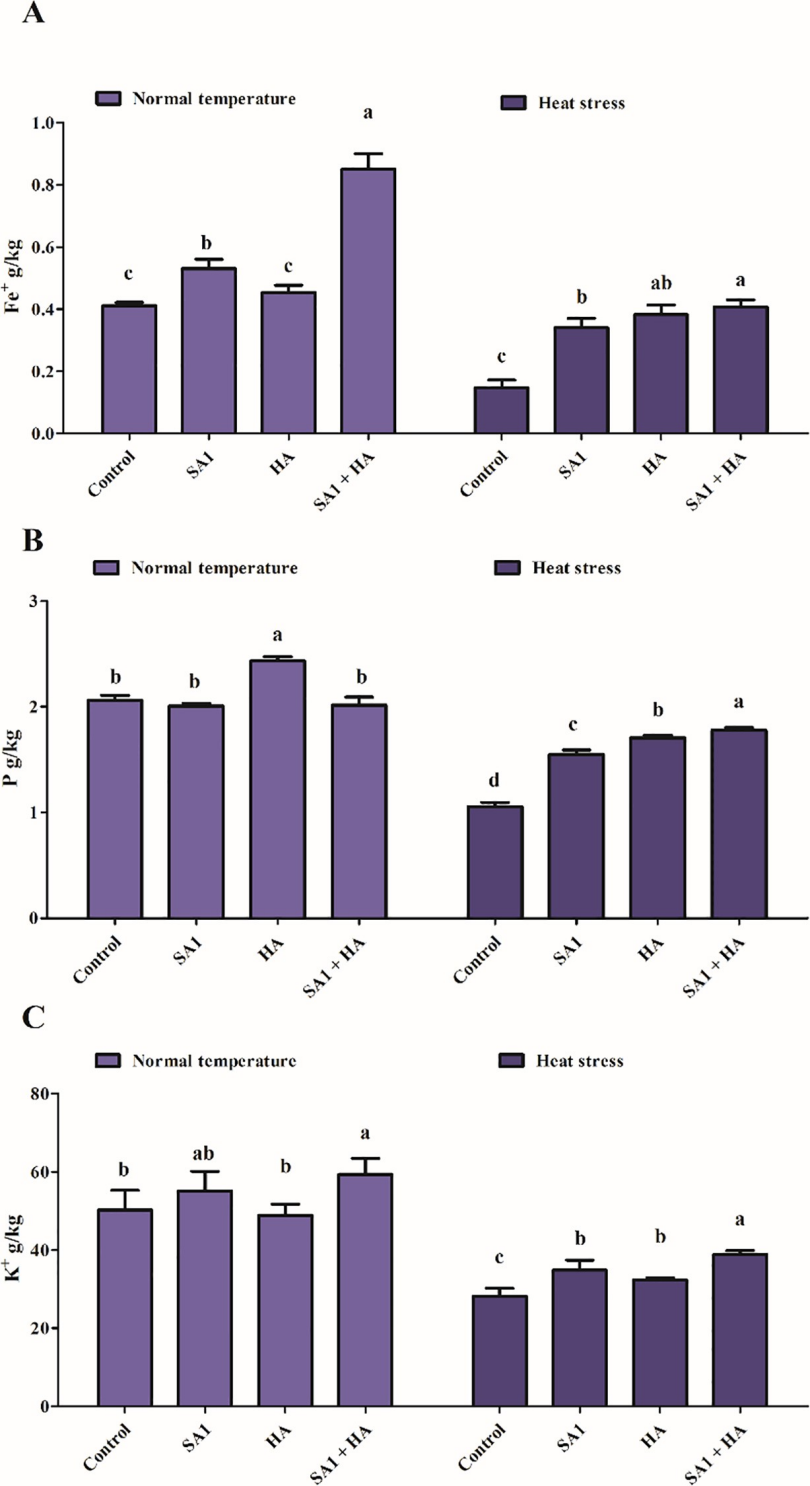
Effect of SA1 an isolate of *Bacillus cereus* (SA1), humic acid (HA) and combined SA1+HA application on ion content; (A) iron (Fe^+^), (B) phosphorus (P), and (C) potassium (K^+^) content in tomato plants under normal and heat stress. Each data point is the mean of three replicates. Error bars represent standard errors. The bars presented with different letters are significantly different from each other as evaluated by Duncan’s Multiple Range Test (DMRT) analysis.

Inductively-coupled plasma mass spectrometry (ICP) results showed that under control conditions, SA1, HA and combined SA1+HA-treated plants had significantly higher amounts of iron (Fe^+^), phosphorus (P) and potassium (K) content compared to the control tomato plants under normal and heat stress conditions (Fig 5). Fe^+^ content revealed a significant difference in tomato plants treated with sole SA1 (28%), HA (6%) and combined SA1+HA (105%) under normal temperature. Heat stress significantly decreased the Fe^+^ content (63%); however, plants treated with SA1 (153%), HA (126%) and combined SA1+HA (158%) had a significantly higher content of Fe^+^ (Fig 5A). Furthermore, P and K content were significantly decreased in heat stressed control plants (49% and 43%). However, bacterial inoculation and HA mitigated heat stress and enhanced the content of P (45–67%) and K (11–34%) (Fig 5B and 5C).

### 3.8. Effect of isolate SA1 and HA on gene expression

**Fig 6:**
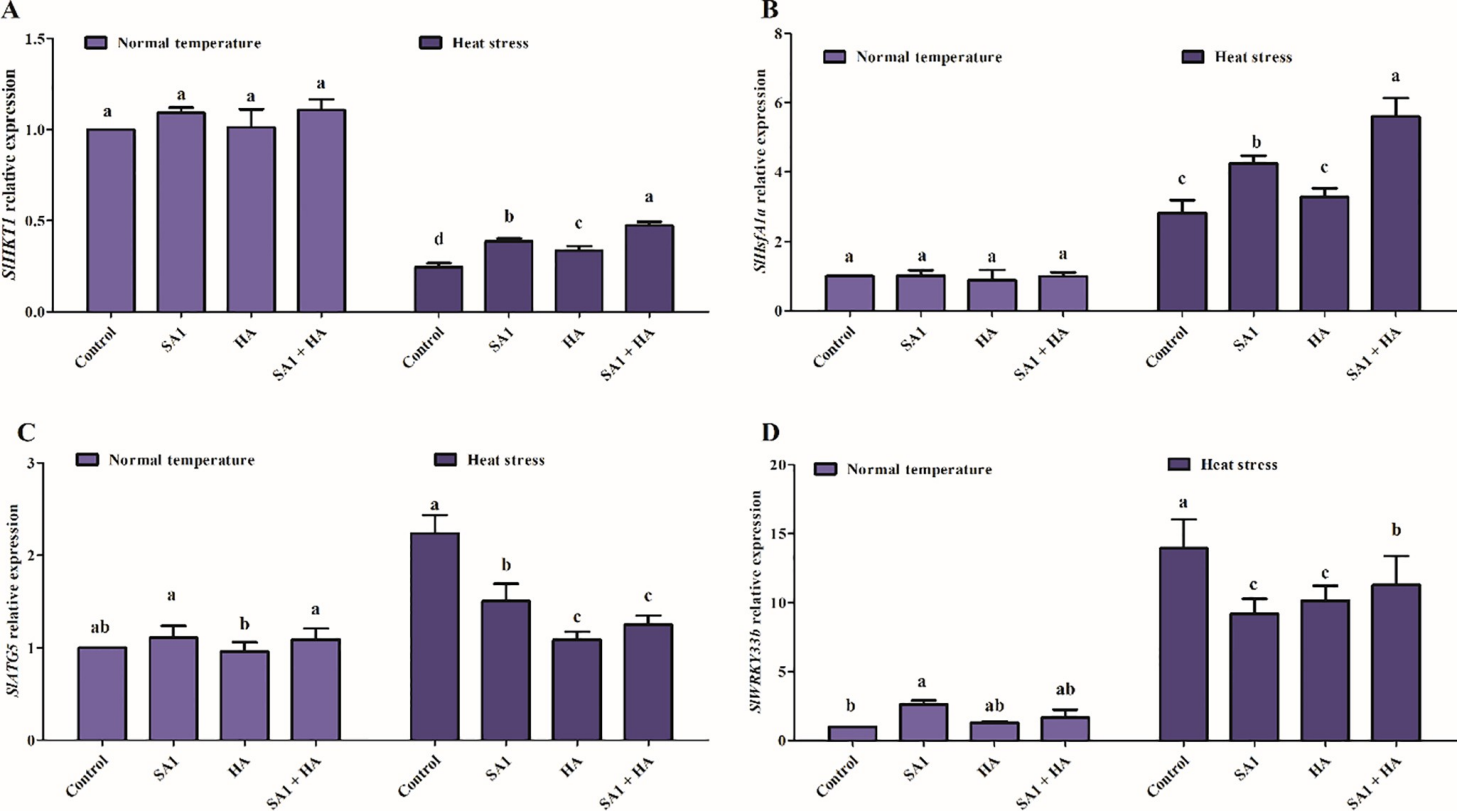
Relative expression of genes in tomato plants treated with SA1, humic acid (HA) and combined SA1+HA under normal and heat stress. (A) *SlHKT1*, (B) *SlHsfA1a*, (C) *SlWRKYY33b* and (D) *SlATG5*. The values were calculated relative to those of actin gene expression and are the means of three replicates. Error bars represent standard errors. The bars presented with different letters are significantly different from each other as evaluated by DMRT analysis.

In response to heat stress, the transcriptional levels of *SlHSFs*, *SlHSPs*, high affinity potassium transporter (*SlHAKT1*), and *SlWRKY33b* were evaluated (Fig 6). *SlHAKT1* gene expression was significantly upregulated in the combined HA and SA1-inoculated tomato plants (10%) compared to the control and sole HA (1.5%) and SA1-treated (9%) plants under normal conditions. Heat stress significantly reduced the expression level of *SlHKT1* (5 fold) in control plants. However, SA1 inoculation and HA mitigated heat stress and enhanced *SlHKT1* gene expression up to 2.5 and 3 fold (Fig 6A). *SlHsfA1a* is one of the master regulators required for induction of the heat stress response. *SlHsfA1a* expression results showed few differences in tomato plants under normal temperature, whereas the *SlHsfA1a* genes were upregulated (2.9-fold). However, combined application of SA1 and HA significantly enhanced the expression of *SlHsfA1a* (2.8 fold) compared to sole SA1 (1.4 fold) and HA (0.3 fold) application and control heat stressed plants (Fig 6B). Furthermore, heat stress-induced autophagy-related genes (ATGs) in the control plants were highly upregulated (1.2 fold) compared to the levels in the control plants. Co-application of SA1 and HA significantly reduced the expression of the *SlATG5* gene (1.15 fold) (Fig 6C). The *SlWRKY33b* transcriptional factor plays a key role in plant stress responses. Our results showed that under normal growth conditions, there is a slight increase in the expression of *SlWRKY33b* genes in control and treated plants. When tomato plants were subjected to heat stress, the overexpression of *SlWRKY33b* genes was observed (13 fold) in control heat stressed plants. However, the application of SA1, HA and combined SA1 and HA significantly mitigated heat stress tolerance and the induced the downregulation of the *SlWRKY33b* genes (2.3–4 fold) (Fig 6D).

### 3.9. Amino acid quantification

Amino acid contents were determined to elucidate the regulation of physiological function with the inoculation of SA1, HA and combined application of SA1+HA in tomato plants under normal and heat stress conditions (Table 2). Under normal conditions, a slight increase was observed in aspartic acid, glutamic acid, alanine, phenylalanine, arginine and proline contents in SA1, HA and combined SA1+HA-treated plants compared with the levels in control plants. Under heat stress, the contents of aspartic acid (59%), glutamic acid (89%), arginine (113%) and proline (67%) were increased, while decreases were observed in alanine (33%) and phenylalanine (28%) contents. However, sole SA1, HA and combined SA1+HA application increased the content of aspartic acid (6, 12 and 10.57%), glutamic acid (18, 14 and 24%), arginine (31, 36 and 89%), alanine (4, 11 and 31%), phenylalanine (6, 28 and 49%) and proline (7, 13 and 3.24%) compared to the levels in un-inoculated tomato plants under heat stress (Table 2). Total amino acid results showed that heat stress markedly decreased the amino acid content up to 48% as compared to the levels in control plants. However, with the application of SA1, HA and SA1+HA, a significant increase in the total amino acid content was observed (85, 12 and 4.34%).

**Table 2:** Amino acids quantification in tomato plants inoculated with SA1 an isolate of B. cereus, humic acid (HA) and SA1+HA under normal and heat stress.

	Aspartic acid	Glutamic acid	Alanine	Phenylalanine	Arginine	Proline
**Normal Temperature**								
**Control**	7.22±0.6^b^	9.55±1.0^b^	8.55±1.1^c^	8.08±1.2^c^	3.51±0.3^b^	7.32±1.1^c^
**SA1**	10.34±1.2^a^	10.46±2.0^a^	9.98±1.0^b^	9.02±0.9^bc^	4.57±0.4^a^	9.78±0.8^ab^
**HA**	10.34±1.1^a^	10.37±1.2^a^	11.21±1.1^a^	10.78±1.3^b^	4.72±0.5^a^	9.09±1.1^b^
**HA+SA1**	10.22±1.8^a^	10.12±1.0^a^	11.68±1.2^a^	12.13±2.0^a^	4.99±0.6^a^	10.22±1.3^a^
**Heat Stress**								
**Control**	11.54±1.9^c^	18.11±2.1^d^	5.73±0.8^c^	5.77±0.5^d^	7.49±1.0^d^	12.26±1.3^c^
**SA1**	12.31±1.4^b^	19.63±2.3^c^	6.0±0.8^b^	6.13±0.7^c^	9.82±1.1^c^	13.23±1.8^b^
**HA**	12.98±1.1^b^	20.67±1.9^b^	6.37±0.9^b^	7.40±1.0b	10.21±1.3^b^	13.92±1.7^b^
**HA+SA1**	13.89±1.8^a^	22.48±3.2^a^	7.51±1.1^a^	8.65±1.3^a^	14.20±1.5^a^	15.10±1.2^a^

Each data point is the mean of at least three replicates. Error bars represent standard errors. The bars presented with different letters are significantly different from each other as evaluated by Duncan’s Multiple Range Test (DMRT) analysis.

## 4. Discussion

Tomato is heat-sensitive and its growth, yield and quality are highly influenced by heat stress. High temperatures cause anatomical, physiological, morphological, and molecular changes in tomato plants, which affect plant growth and consequently affect plant yield. Presently, tomato is widely consumed raw or industrially processed and is considered a good source of nutritional properties and its different beneficial pigment constituents have paved the way for increased demand all over the world. Therefore, there is a need to boost tomato production under heat stress conditions. Consequently, substantial efforts have been extended to the use of physiochemical and ecofriendly biological approaches, which are currently being applied to alleviate plant heat stress. Recently, the co-administration of HA and the *Bacillus* genus as important plant bio-stimulants that improve the growth and productivity in different crops [41,49] while reducing the dependency on chemical fertilizers has begun to gain importance. Previous reports demonstrated that humic substances may favor bacteria-plant interactions and enhance the bacterial attachment and colonization [47,74]. Olivares et al. [40] and Canellas et al. [75] recently reported the beneficial effects of combined application of heat stress and PGPB on various crops like maize and tomato. These findings were consistent with the present results that tomato plants treated with isolate SA1+HA enhance root/shoot length, biomass and chlorophyll content under normal and heat stress condition (Table 1; Fig 2). Among the physiological activities of plants, photosynthesis is the most sensitive to heat stress because in the chloroplast, the stroma is severely affected by increased temperature [11–13,76–78]. Many plants species have evolved several mechanisms to guard the photosynthetic apparatus against heat stress damages by encoding for the production of HSPs that binds to thylakoid membranes and protect PSII and the electron transport chain [76,78–80]. However, under severe heat stress, these protective mechanisms may be inadequate to ensure plant viability. Our findings showed that an increase of chlorophyll florescence in treated tomato plants occurred under normal and heat stress conditions compared to un-inoculated tomato plants (Table 1). Similar results were also observed by other researchers [26,81], who reported that the use of thermotolerant agents increased total chlorophyll content in wheat and canola plants under heat stress. Enhanced chlorophyll content may also be the result of increased photosynthetic leaf area due to the application of microbes and HA compared to the control (stressed) plants [26,27]. This increase in chlorophyll content is probably due to enhanced moisture retention and the improvement of nutrient supply in the root zone. Canellas et al. [75] reported that co-application of PGPB and heat stress increased the net photosynthetic rate and nutrient uptake, which ultimately led to vigorous and healthy crop growth and productivity.

K, P and Fe^+^ are important elements required for plant growth and function regulation in several biochemical processes like protein synthesis, enzymatic activity and hormonal regulation [82–86]. Several physiological processes depend on Fe, K and P such as photosynthesis, stomatal regulation and abiotic stress tolerance. Under abiotic stress, K helps to maintain ion homeostasis, regulate osmatic balance and stomatal opening and enhance the antioxidant defense system in plants [87,88]. Baldotto et al. [89] revealed that pineapple plant growth was affected by *Burkholderia* strain inoculation and HAs increased the root and shoot growth, biomass and nutrient contents (nitrogen [N] 132%, P 131% and K 80%) compared to un-inoculated plants. These results are consistent with findings in the present study that tomato plants treated with SA1+HA exhibited enhanced P, K and Fe content under normal and heat stress condition (Fig 5). Previously, Schoebitz et al. [90] reported that combined administration of PGP microbes and HA increased N and K uptake and growth in blueberry plants compared to the control plants.

Phytohormones greatly respond to changing environmental conditions by regulating plant growth and are actively involved in the response to heat stress [91–93]. IAA is a key phytohormone that plays a key role in the growth, development and tolerance of plants to heat stress through the activation of antioxidant enzymes, regulation of gene expression, synthesis of osmoprotectants (proline), and enhanced accumulation of photosynthetic pigments [94]. It has been reported that IAA-producing bacteria increase the length and root surface of plants, helping the plants get better access to nutrients available in the soil [24,95]. Our bacterial isolate (SA1) was also capable of producing IAA and GAs (Fig 1A and 1B) and greatly mitigated the adverse effects of heat stress on tomato plants. Additionally, GAs play a prominent role in the alleviation of abiotic stress [96,97]. Stavang et al. [98] reported that GAs play a vital role in alleviating heat stress and enhanced growth in *Arabdopasis* and pea. Microbes that produced GAs are important for promoting plant growth and mitigating the adverse effects of heat stress [24,98,99]. Despite the existence of several forms of GA, biologically active GA is limited in different microorganisms [100,101]. The microbes used in our study produce bioactive GAs (Fig 1B). These biologically active GAs promote plant growth through reducing stress hormones like ABA [102–104]. When plants perceive stressful conditions, they regulate stress hormones like ABA through active chemical signals, which induce extreme sensitivity to stomatal conductance [104,105]. The prolific role of plant microbe interaction lies in mitigating the adverse effects of abiotic stress by reducing ABA content [24,25,106]. Similar results were observed in our study in which application of isolate SA1 and HA caused a decrease in ABA content and an increase in plant growth parameters (Fig 3A). Salicylic acid (SA) is another plant hormone that plays an important role in various physiological processes and biochemical reactions [107]. According to Zhang et al. [108] and Khan et al [107], in mutualistic plant-microbe interactions, SA induces systemic resistance in plants. Wang et al. [109] and Khan et al [107] suggested that SA can ameliorate abiotic stress by inducing ROS generation. The accumulation of SA has also been implicated in heat stress tolerance in various plants including Kentucky Bluegrass [23], grapevine [110], potato [111], bean and tomato [112], Arabidopsis [113] and grape plants [114]. Isolate SA1+HA application greatly enhanced the endogenous SA level in tomato plants under heat stress and normal conditions (Fig 3B). Our findings are in agreement with previous reports in which the ability of bacterial inoculum enhanced the endogenous levels of SA and contributed to the growth and development of plants under abiotic stress conditions [115,116].

When plants are subjected to environmental stress including heat stress, a variety of ROS are generated [117,118]. These ROS interfere with different organic molecules, resulting in the reduction of photosynthesis and subsequently reduced plant growth [117,119]. Under heat stress, plants are capable of counteracting ROS generation by the production of different antioxidant molecules (enzymatic and non-enzymatic) [117,118]. The key antioxidant enzymes that play an effective role in scavenging ROS are SOD, catalase (CAT), and peroxidase [120,121]. The activities of these enzymes usually increase under stress conditions [85,86,122–125]. Tomato plants treated with isolate SA1 and HA produced less ROS and showed an increase in antioxidant activities like SOD, MDA, GSH, and APX compared with un-inoculated plants under heat stress (Fig 4). Similar results were reported in previous studies [24,27,33,121,126], where PGPB enhanced the activity of different ROS scavenging enzymes under heat stress. Amino acids play a prominent role in the physiological and biochemical functions of plants such as the modulation of membrane permeability, osmolytes, ion uptake, enzymatic activity and tolerance to abiotic stress [127,128]. Amino acids also act as bio-stimulants for the growth attributes of plants and significantly mitigate injuries caused by abiotic stresses [121]. The co-application of SA1+HA exhibited a rescue effect and significantly enhanced the important endogenous amino acid content under heat stress compared with control heat stressed plants (Table 2). Several previous reports indicated that an increase in amino acid content increases tolerance to abiotic stress following inoculation with plant growth-promoting bacteria [120,129–133]. Heat stress reduced the accumulation of proline, but proline levels were enhanced in SA1+HA treated plants under control and heat stress conditions (Table 2). Similarly, proline accumulation is considered an adaptive mechanism under heat stress [134–137].

Plants contain several heat-stress-dependent genes such as HSFs and HSPs that enhance tolerance and protect the function of proteins under heat stress conditions. HSFs are essential for maintaining and restoring protein structure as well as stabilizing the condition of plants under heat stress [19,138,139]. Fragkostefanakis et al. [140] reported the upregulation of HSF level in bacteria-inoculated wheat seedlings during heat stress as well as a decrease in the HSP transcript levels. Similar *SlHsfA1a* transcriptional levels were observed in sole SA1, HA and combined SA1+HA application in tomato plants under heat stress (Fig 6). The induction of HSPs is usually accompanied by tolerance to heat and other stresses. Previous studies revealed that HSFs act as “molecular chaperones” and the over-expression of these genes and proteins is well established to enhance thermotolerance [18,20,141]. Similarly, Sl*WRKY33b*, *SlHAKT1* and *SlATG5* proteins and play critical roles in tomato heat tolerance. *HKT1* plays a crucial role and higher cellular K concentration during stress is critical for normal plant function [142]. We observed higher expression of the *SlHKT1* gene in sole SA1, HA and combined SA1+HA-treated plants under normal and heat stress conditions. Ali et al. [143], Fairbairn [144] and Almeida [142] reported that higher expression of the *SlHKT1* gene enhances stress tolerance and the accumulation of K content. Furthermore, based on previous reports, we evaluated the expression of *SlWRKY33b* and *SlATG5* and found that both genes were highly expressed in plants grown under heat stress (37°C), indicating that both of these genes are highly correlated with heat stress. However, plants treated with bacteria and HA under the same degree of heat stress showed comparatively less regulation of both genes, suggesting that bacteria and HA also have key roles in heat tolerance. Our study was in agreement with that of Zhou [145], who reported that heat stress significantly induced the regulation of the *SlATG* gene and *SlWRKY33*, which physically interacts with the autophagy-related protein. Other reports demonstrated that silencing the *SlWRKY33* gene in tomato reduces *SlATG* gene expression, which compromises tomato heat tolerance [146].

## 5. Conclusions

In conclusion, the present study demonstrated that isolate SA1 has the ability to produce biologically active metabolites such as GA, IAA and organic acids. Tomato plants treated with isolate SA1 and HA showed significant improvements in their growth attributes and chlorophyll fluorescence under normal and heat stress conditions. This improvement in plant growth was coupled with changes in endogenous phytohormones (ABA and SA), antioxidants (APX, SOD, GSH and LPO), and essential amino acids and the expression of genes such as *SlWRKY33b*, *SlHKT1* and *SlATG5*. The stability and increased consistency of the tomato plant response to bacterial inoculation in the presence of HA indicated a promising co-friendly biological approach and physicochemical biotechnological tool to improve the growth and development of tomato under heat stress conditions.

## Supporting information

S1 Table List of primers used. (DOCX)

S2 Table Description of plants species and number of their yielded endophytic isolates. The isolates were preliminary sorted for single or multiple plant growth beneficial activities.
(DOCX)

S1 Fig Multiple plant growth-promoting traits-producing bacteria were grown in LB media at 25°C, 30°C, 35°C, 40°C and 45°C for 6 days and the growth was recorded using a spectrophotometer at 600 nm. Each data point is the mean of three replicates.
(DOCX)

S2 Fig Isolate SA1 were grown in LB media at 25°C, 30°C, 35°C, 40°C, and 45°C for 36 hours, and the growth was examined using a spectrophotometer at 600 nm. Each data point is the mean of three replicates.
(DOCX)

S3 Fig Phylogenetic tree based on 16S rRNA sequences of endophytic bacterial strain SA1 isolated from the roots of *Echinochloa crus-galli* (L.) Beauv plant. (DOCX)

## Abbreviations

PGPEB: plant growth-promoting endophytic bacteria
GA: gibberellin
IAA: indole-3-acetic acid
ABA: abscisic acid
SA: salicylic acid
APX: ascorbic acid peroxidase
SOD: superoxide dismutase
GSH: glutathione
HSF: heat shock transcription factor
*SlHKT1*: potassium channel *HKT1*
