# Supplementary material for: Extending thermotolerance to tomato seedlings by inoculation with SA1 isolate of Bacillus cereus and comparison with exogenous humic acid application
Source: PLoS One. 2020 Apr 30;15(4):e0232228. doi: 10.1371/journal.pone.0232228 (PMC7192560; doi:10.1371/journal.pone.0232228)
Supplement: S1 Table — (DOCX) [file pone.0232228.s001.docx]

**S1 Table 1. List of primers used.**

| **Primers** | | |
| --- | --- | --- |
| ***SlHsfA1a*** | F | 5̕ TTGGCGCGCCATGGAGCCGAATTCTTAT3̕ |
|  | R | 5̕ GGGGTACCGATCATATGTTTTTGTTG3̕ |
| ***SlWRKY33b*** | F | 5̕ GCATTACTGTCAACCATCGC3̕ |
|  | R | 5̕ AACTTCGCGGATTCTCACTT3̕ |
| ***SlATG5*** | F | 5̕ TCAGATGGTGCTGAGATCAAG3̕ |
|  | R | 5̕ ATTGTTTACCACCCATGCAA3̕ |
| ***SlHKT1*** | F | 5̕ CCTAGAACCCTACCGTC3̕ |
|  | R | 5̕ GAACATTTGAGAAAACTTCC3̕ |
| ***Actin*** | F | 5̕ TGTCCCTATTTACGAGGGTTATGC3̕ |
|  | R | 5̕ CAGTTAAATCACGACCAGCAAGAT3̕ |
