# Supplementary material for: Extending thermotolerance to tomato seedlings by inoculation with SA1 isolate of Bacillus cereus and comparison with exogenous humic acid application
Source: PLoS One. 2020 Apr 30;15(4):e0232228. doi: 10.1371/journal.pone.0232228 (PMC7192560; doi:10.1371/journal.pone.0232228)
Supplement: S2 Table — The isolates were preliminary sorted for single or multiple plant growth beneficial activities. (DOCX) [file pone.0232228.s002.docx]

**S2 Table. Description of plants species and number of their yielded endophytic isolates.** The isolates were preliminary sorted for single or multiple plant growth beneficial activities.

| **Plants name** | **No. of isolates** | **Isolates having single Plant Growth Promoting characteristics** | | | **Isolates with multiple PGP characteristics** |
| --- | --- | --- | --- | --- | --- |
|  |  | **IAA production** | **Siderophore** | **Phosphate** |  |
| **Endophytes isolates** | | | | | |
| *Artemisia princeps* Pamp. | 24 | 16 | 5 | 6 | 8 |
| *Chenopodium ficifolium* Smith. | 6 | 1 | 0 | 2 | 0 |
| *Oenothera biennis* L. | 17 | 12 | 1 | 2 | 1 |
| *Echinochloa crus-galli* (L.) Beauv. | 12 | 7 | 1 | 3 | 4 |
