# Supplementary material for: Extending thermotolerance to tomato seedlings by inoculation with SA1 isolate of Bacillus cereus and comparison with exogenous humic acid application
Source: PLoS One. 2020 Apr 30;15(4):e0232228. doi: 10.1371/journal.pone.0232228 (PMC7192560; doi:10.1371/journal.pone.0232228)

**Supplementary figure 1.**

Multiple plant growth-promoting traits-producing bacteria were grown in LB media at 25ºC, 30ºC, 35ºC, 40ºC and 45ºC for 6 days and the growth was recorded using a spectrophotometer at 600 nm. Each data point is the mean of three replicates.


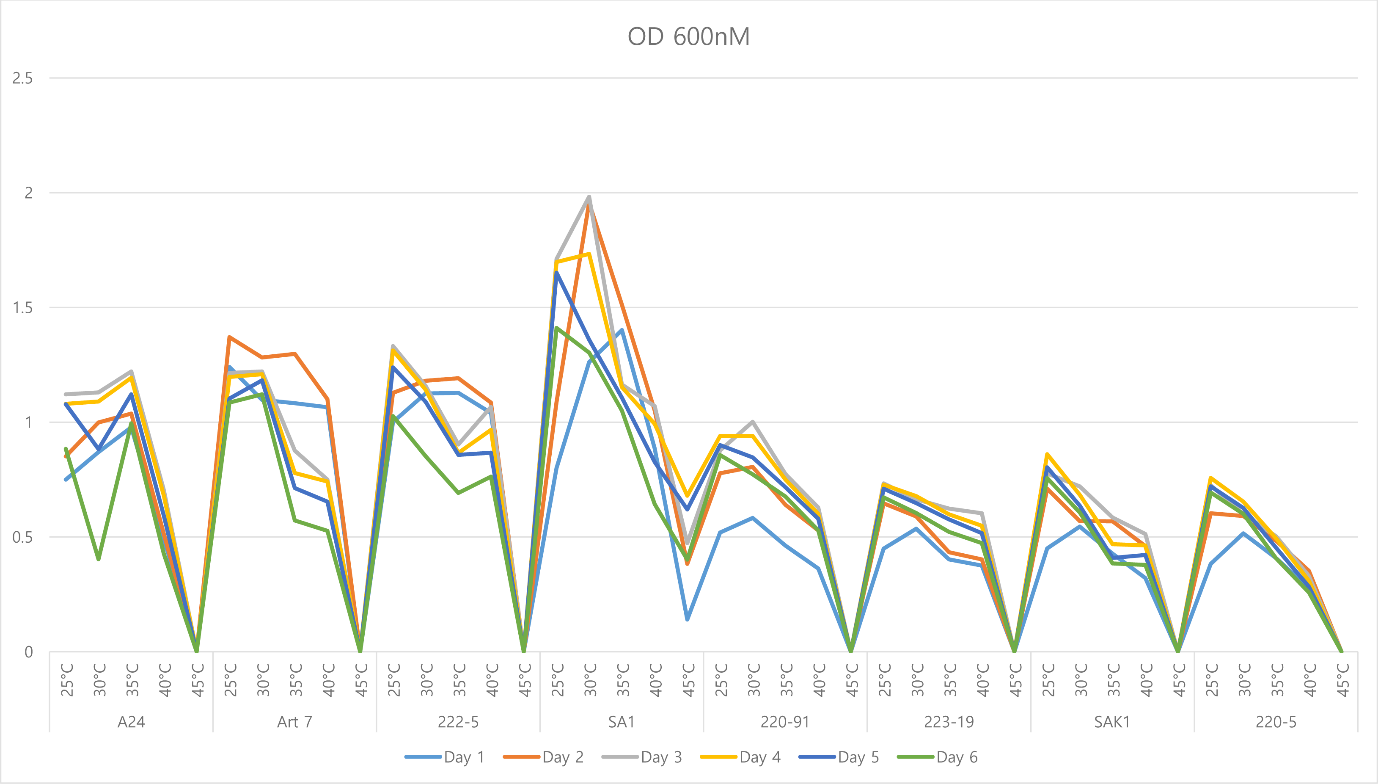

Supplement: S1 Fig — Each data point is the mean of three replicates. (DOCX) [file pone.0232228.s003.docx]
