# Supplementary material for: Extending thermotolerance to tomato seedlings by inoculation with SA1 isolate of Bacillus cereus and comparison with exogenous humic acid application
Source: PLoS One. 2020 Apr 30;15(4):e0232228. doi: 10.1371/journal.pone.0232228 (PMC7192560; doi:10.1371/journal.pone.0232228)
Supplement: S2 Fig — Each data point is the mean of three replicates. (DOCX) [file pone.0232228.s004.docx]

**Supplementary figure 2.**

Isolate SA1 were grown in LB media at 25°C, 30°C, 35°C, 40°C, and 45°C for 36 hours, and the growth was examined using a spectrophotometer at 600 nm. Each data point is the mean of three replicates.

**
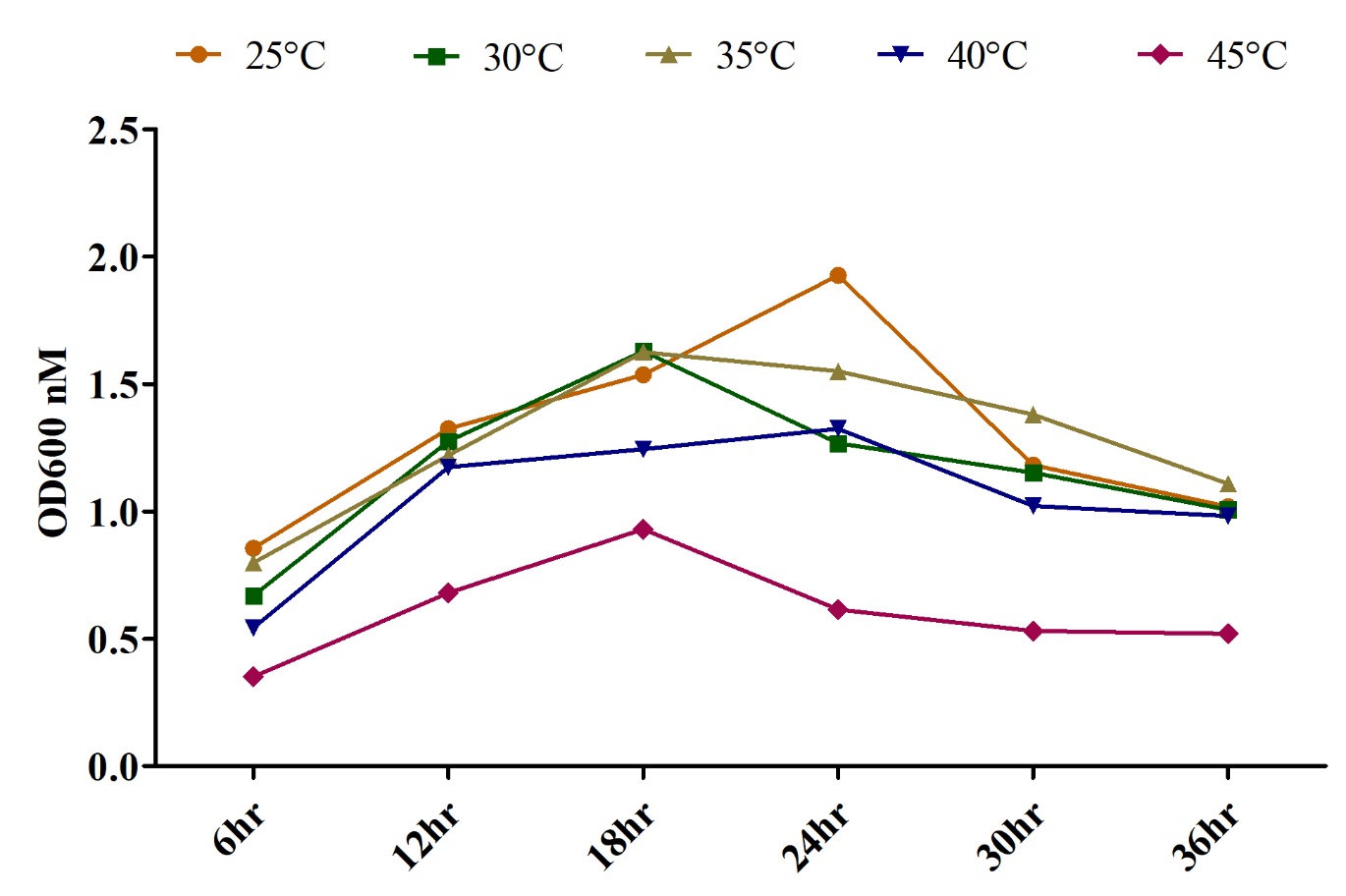
**
