# Supplementary material for: Extending thermotolerance to tomato seedlings by inoculation with SA1 isolate of Bacillus cereus and comparison with exogenous humic acid application
Source: PLoS One. 2020 Apr 30;15(4):e0232228. doi: 10.1371/journal.pone.0232228 (PMC7192560; doi:10.1371/journal.pone.0232228)

**Supplementary figure 3.**

Phylogenetic tree based on 16S rRNA sequences of endophytic bacterial strain SA1 isolated from the roots of *Echinochloa crus-galli* (L.) Beauv plant.


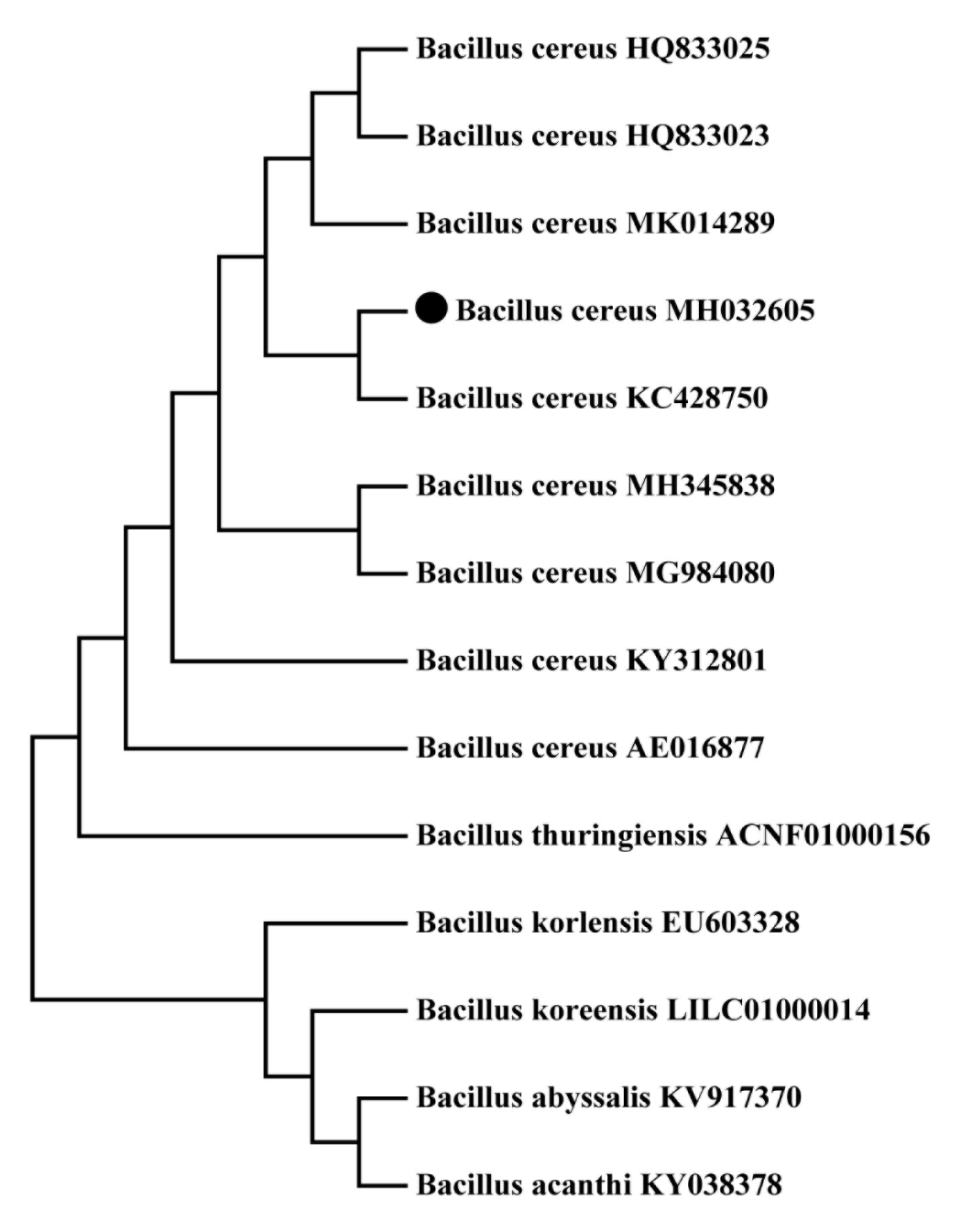

Supplement: S3 Fig — (DOCX) [file pone.0232228.s005.docx]
